# Supplementary material for: Runs of Homozygosity Revealed Reproductive Traits of Hu Sheep
Source: Genes (Basel). 2022 Oct 13;13(10):1848. doi: 10.3390/genes13101848 (PMC9601733; doi:10.3390/genes13101848)
Supplement: Supplementary file 1 [file genes-13-01848-s001.zip › genes-1953592-supplementary.pdf]

Supplementary Material

Runs of Homozygosity Revealed Agriculture Characteristics of Hu Sheep

Yuzhe Li[1,2], Zitao Chen[2], Yifei Fang[3], Caiyun Cao[1,2], Zhe Zhang[1], Yuchun Pan[1,2], Qishan Wang[1,2]*

[1] Hainan Institute, Zhejiang University, Yongyou Industry Park, Yazhou Bay Sci-Tech City, Sanya 572000, China

[2] Department of Animal Science, College of Animal Science, Zhejiang University, 866# Yuhangtang Road, Hangzhou, East 310058, China.

[3] Department of Animal Science, School of Agriculture and Biology, Shanghai Jiao Tong University, 800# Dongchuan Road, Shanghai, East 200240, China.

[*] Correspondence: Qishan Wang

Email: wangqishan@zju.edu.cn

Table S1. Quality of Tn5-based low-coverage sequencing among 108 Hu sheep.

Sample Name	Depth	Coverage	Reads
YH-Sheep-1	1.30	0.29	14470004
YH-Sheep-2	1.27	0.21	9580172
YH-Sheep-3	1.32	0.26	11416834
YH-Sheep-4	1.31	0.30	15410636
YH-Sheep-5	1.24	0.24	12016190
YH-Sheep-6	1.28	0.27	13422318
YH-Sheep-7	1.25	0.26	15753402
YH-Sheep-8	1.25	0.23	9824162
YH-Sheep-9	1.28	0.25	10235936
YH-Sheep-10	1.28	0.25	11096436
YH-Sheep-18	1.32	0.27	12465638
YH-Sheep-19	1.31	0.28	16155766
YH-Sheep-20	1.34	0.35	16040168
YH-Sheep-41	1.25	0.28	12842938
YH-Sheep-51	1.27	0.29	12759120
YH-Sheep-52	1.18	0.21	8505194
YH-Sheep-53	1.22	0.21	9515366
YH-Sheep-54	1.35	0.32	14567264
YH-Sheep-55	1.27	0.25	11048140
YH-Sheep-56	1.35	0.36	16122268
YH-Sheep-57	1.24	0.22	8965962
YH-Sheep-58	1.24	0.23	9336530
YH-Sheep-59	1.17	0.17	6693776
YH-Sheep-62	1.22	0.20	8000548
YH-Sheep-63	1.21	0.17	7507686
YH-Sheep-64	1.20	0.18	7098036
YH-Sheep-65	1.19	0.19	7613942
YH-Sheep-66	1.23	0.20	8097650
YH-Sheep-67	1.20	0.18	8399238
YH-Sheep-68	1.21	0.20	11911472
YH-Sheep-69	1.29	0.28	10346418
YH-Sheep-70	1.26	0.27	13522470
YH-Sheep-71	1.32	0.32	12700952
YH-Sheep-72	1.28	0.29	17281534
YH-Sheep-73	1.37	0.39	12763108
YH-Sheep-74	1.31	0.29	13891984
YH-Sheep-75	1.31	0.32	16783722
YH-Sheep-76	1.35	0.37	12220166
YH-Sheep-77	1.28	0.29	10619462
YH-Sheep-78	1.32	0.27	14257506
YH-Sheep-79	1.31	0.32	11850188

Sample Name	Depth	Coverage	Reads
YH-Sheep-80	1.28	0.29	10527508
YH-Sheep-81	1.26	0.26	11651424
YH-Sheep-82	1.28	0.28	11939012
YH-Sheep-83	1.28	0.29	15659692
YH-Sheep-84	1.36	0.37	13875886
YH-Sheep-85	1.29	0.32	11653342
YH-Sheep-86	1.25	0.22	17257720
YH-Sheep-87	1.35	0.36	17257720
YH-Sheep-88	1.33	0.35	15983072
YH-Sheep-89	1.28	0.31	14075620
YH-Sheep-91	1.35	0.36	16417264
YH-Sheep-93	1.31	0.34	15473524
YH-Sheep-95	1.30	0.35	15685220
YH-Sheep-97	1.26	0.30	15687654
YH-Sheep-98	1.25	0.28	11398732
YH-Sheep-99	1.29	0.34	10064142
YH-Sheep-101	1.28	0.32	14923448
YH-Sheep-102	1.28	0.33	13645110
YH-Sheep-103	1.29	0.34	13176506
YH-Sheep-104	1.29	0.32	14029150
YH-Sheep-105	1.31	0.36	14907622
YH-Sheep-106	1.27	0.31	15725108
YH-Sheep-107	1.30	0.33	13418390
YH-Sheep-108	1.30	0.35	15052138
YH-Sheep-110	1.24	0.29	14002870
YH-Sheep-111	1.28	0.33	13112016
YH-Sheep-112	1.26	0.28	11923470
YH-Sheep-113	1.29	0.31	12948080
YH-Sheep-114	1.27	0.30	11250040
YH-Sheep-115	1.28	0.32	12741250
YH-Sheep-116	1.30	0.32	13009600
YH-Sheep-117	1.29	0.33	12554526
YH-Sheep-118	1.25	0.29	11636622
YH-Sheep-119	1.27	0.28	10084760
YH-Sheep-120	1.25	0.29	10539244
YH-Sheep-121	1.32	0.34	14115538
YH-Sheep-122	1.34	0.37	16269724
YH-Sheep-123	1.30	0.30	12201158
YH-Sheep-124	1.35	0.34	14207752
YH-Sheep-125	1.31	0.27	10608088
YH-Sheep-126	1.36	0.36	16076862
YH-Sheep-127	1.31	0.32	13145590
YH-Sheep-128	1.33	0.31	14166404

Sample Name	Depth	Coverage	Reads
YH-Sheep-129	1.31	0.32	13531778
YH-Sheep-130	1.36	0.38	12701288
YH-Sheep-131	1.30	0.31	12680966
YH-Sheep-132	1.32	0.31	11755996
YH-Sheep-133	1.27	0.29	11043064
YH-Sheep-134	1.26	0.27	11712110
YH-Sheep-135	1.28	0.29	11229422
YH-Sheep-136	1.28	0.28	11312464
YH-Sheep-137	1.29	0.29	10515380
YH-Sheep-138	1.26	0.26	11732656
YH-Sheep-139	1.29	0.29	13674258
YH-Sheep-140	1.36	0.33	11785346
YH-Sheep-141	1.28	0.29	10296560
YH-Sheep-142	1.27	0.26	12498262
YH-Sheep-143	1.29	0.29	11262522
YH-Sheep-144	1.28	0.28	11904870
YH-Sheep-145	1.29	0.28	12953392
YH-Sheep-146	1.30	0.30	12226238
YH-Sheep-147	1.29	0.30	12250158
YH-Sheep-148	1.37	0.33	14672722
YH-Sheep-149	1.32	0.33	13795518
YH-Sheep-150	1.29	0.31	13666194
YH-Sheep-151	1.30	0.31	14687360
YH-Sheep-153	1.33	0.34	13666194

Table S2. List of 128 potential candidate genes within ROH islands in the Hu sheep.

Chr	Gene Start (bp)	Gene End (bp)	Gene Name	Ensembl Gene ID
1	27288843	27325319	*RNF11*	ENSOARG00020004026
2	1.23E+08	1.23E+08	*LGSN*	ENSOARG00020015419
2	1.23E+08	1.23E+08	*OCA2*	ENSOARG00020015487
2	1.23E+08	1.23E+08	*HERC2*	ENSOARG00020015875
3	1.13E+08	1.13E+08	*ZC3H8*	ENSOARG00020001922
3	1.13E+08	1.13E+08	*ZC3H6*	ENSOARG00020001977
5	45548809	45558086	*R3HDM4*	ENSOARG00020012831
5	45561363	45564709	*KISS1R*	ENSOARG00020012927
5	45577442	45605740	*ARID3A*	ENSOARG00020013070
5	45612574	45617592	*WDR18*	ENSOARG00020013193
5	45622020	45631253	*GRIN3B*	ENSOARG00020013299
5	45631701	45638037	*TMEM259*	ENSOARG00020013430
5	45671806	45684186	*ARHGAP45*	ENSOARG00020014441
5	45701349	45728657	*SBNO2*	ENSOARG00020015016
5	45763394	45779202	*STK11*	ENSOARG00020015219
5	45780799	45786768	*CBARP*	ENSOARG00020015344
5	45791439	45793371	*ATP5F1D*	ENSOARG00020015444
5	45816115	45823436	*CIRBP*	ENSOARG00020015562
5	45821461	45824684	*FAM174C*	ENSOARG00020015682
5	45831412	45844715	*EFNA2*	ENSOARG00020015712
5	45894462	45907926	*PWWP3A*	ENSOARG00020015787
5	45915035	45920824	*NDUFS7*	ENSOARG00020015840
5	45921243	45924269	*GAMT*	ENSOARG00020015902
5	45931282	45949747	*DAZAP1*	ENSOARG00020015974
5	52061481	52096528	*CDC25C*	ENSOARG00020014762
5	52106512	52109997	*FAM53C*	ENSOARG00020015352
5	52115380	52173526	*KDM3B*	ENSOARG00020015437
5	52176357	52181066	*REEP2*	ENSOARG00020015925
5	52263951	52278149	*HSPA9*	ENSOARG00020016252
5	52278568	52610584	*CTNNA1*	ENSOARG00020016402
5	52548172	52550074	*LRRTM2*	ENSOARG00020016498
5	52619483	52886681	*SIL1*	ENSOARG00020016552
5	52992040	53008425	*PAIP2*	ENSOARG00020017952
5	53007611	53021506	*SLC23A1*	ENSOARG00020018006
5	53022713	53024812	*MZB1*	ENSOARG00020018100
5	53045014	53070802	*DNAJC18*	ENSOARG00020018261
5	53088964	53090746	*SMIM33*	ENSOARG00020018356
5	53094862	53099768	*STING1*	ENSOARG00020018383
6	33557755	33977806	*UNC5C*	ENSOARG00020019727

Chr	Gene Start (bp)	Gene End (bp)	Gene Name	Ensembl Gene ID
6	33990928	34214488	*BMPR1B*	ENSOARG00020020206
6	34541043	34774247	*PDLIM5*	ENSOARG00020020424
6	34910771	34926294	*HPGDS*	ENSOARG00020020743
10	37362294	37388504	*FGF9*	ENSOARG00020004253
10	37445771	37497434	*MICU2*	ENSOARG00020004275
10	37505860	37562509	*ZDHHC20*	ENSOARG00020004328
10	37585964	37590661	*SAP18*	ENSOARG00020004477
10	37640815	37663713	*LATS2*	ENSOARG00020004530
10	37676610	37763326	*XPO4*	ENSOARG00020004563
10	37770987	37789871	*EEF1AKMT1*	ENSOARG00020004604
10	37790795	37821687	*IL17D*	ENSOARG00020004623
10	37826129	37881608	*IFT88*	ENSOARG00020004679
10	37902431	37961104	*CRYL1*	ENSOARG00020004947
10	38031596	38032381	*GJB6*	ENSOARG00020005011
10	38083322	38084560	*GJA3*	ENSOARG00020005065
10	38101146	38164431	*ZMYM2*	ENSOARG00020005084
10	38215822	38232515	*ZMYM5*	ENSOARG00020005220
10	38243213	38292550	*PSPC1*	ENSOARG00020005341
10	38319717	38365729	*MPHOSPH8*	ENSOARG00020005393
10	38617017	38637015	*ATP12A*	ENSOARG00020006165
11	35721278	35730729	*ALOX15B*	ENSOARG00020022867
11	35752673	35763575	*GUCY2D*	ENSOARG00020023345
11	35801751	35815622	*CNTROB*	ENSOARG00020023412
11	35816656	35817885	*TRAPPC1*	ENSOARG00020023446
11	35834681	35858685	*CHD3*	ENSOARG00020023540
11	35906256	36008814	*DNAH2*	ENSOARG00020010512
11	36018253	36022202	*EFNB3*	ENSOARG00020011424
11	36024266	36035913	*WRAP53*	ENSOARG00020011477
11	36037234	36050137	*TP53*	ENSOARG00020011609
11	36057127	36061394	*ATP1B2*	ENSOARG00020011738
11	36075241	36080260	*SHBG*	ENSOARG00020011830
11	36089500	36102013	*FXR2*	ENSOARG00020012154
11	36101339	36109068	*MPDU1*	ENSOARG00020012323
11	36110820	36112834	*CD68*	ENSOARG00020012774
11	36113791	36189828	*EIF4A1*	ENSOARG00020012888
11	36120652	36128210	*SENP3*	ENSOARG00020013096
11	36130780	36132620	*TNFSF13*	ENSOARG00020013222
11	36134324	36141723	*TNFSF12*	ENSOARG00020013736
11	36180823	36200737	*POLR2A*	ENSOARG00020013804
11	36213780	36218033	*ZBTB4*	ENSOARG00020014211
11	36222232	36230447	*CHRNB1*	ENSOARG00020014250
11	36247152	36248967	*SPEM2*	ENSOARG00020014676

Chr	Gene Start (bp)	Gene End (bp)	Gene Name	Ensembl Gene ID
11	36253944	36339436	*EIF5A*	ENSOARG00020014758
11	36255150	36264793	*NLGN2*	ENSOARG00020014909
11	36276073	36280070	*PLSCR3*	ENSOARG00020015038
11	36281191	36287396	*TNK1*	ENSOARG00020015110
11	36302296	36314335	*ACAP1*	ENSOARG00020015243
11	36331590	36333801	*GPS2*	ENSOARG00020017230
11	36364136	36368808	*SLC2A4*	ENSOARG00020017448
11	36381720	36383714	*CLDN7*	ENSOARG00020017686
11	36390277	36396534	*CTDNEP1*	ENSOARG00020017866
11	36394612	36403886	*GABARAP*	ENSOARG00020017939
11	36401088	36405210	*PHF23*	ENSOARG00020018070
11	36406199	36413251	*DVL2*	ENSOARG00020018284
11	36414027	36421963	*ACADVL*	ENSOARG00020018512
11	36420278	36443824	*DLG4*	ENSOARG00020019051
13	52690746	52753392	*HAO1*	ENSOARG00020003549
13	52972788	52996005	*ADRA1D*	ENSOARG00020003607
13	55947893	55966102	*PCMTD2*	ENSOARG00020009092
13	55981340	56016688	*MYT1*	ENSOARG00020009211
13	56079358	56085713	*OPRL1*	ENSOARG00020009569
13	56096150	56101676	*RGS19*	ENSOARG00020009662
13	56103189	56110487	*TCEA2*	ENSOARG00020009690
13	56130229	56132356	*C20orf204*	ENSOARG00020009776
13	56134569	56169865	*PRPF6*	ENSOARG00020009838
13	56180219	56185755	*ZNF512B*	ENSOARG00020010099
13	56209667	56212074	*DNAJC5*	ENSOARG00020010877
13	56240589	56255424	*TPD52L2*	ENSOARG00020010989
13	56298871	56326994	*ZBTB46*	ENSOARG00020011728
13	56347151	56352866	*ARFRP1*	ENSOARG00020012087
13	56379608	56387570	*STMN3*	ENSOARG00020003323
13	56401600	56412293	*GMEB2*	ENSOARG00020003375
13	56417215	56418216	*FNDC11*	ENSOARG00020003472
13	56420892	56426769	*SRMS*	ENSOARG00020003498
13	56429472	56435767	*PTK6*	ENSOARG00020003662
13	56453555	56461060	*EEF1A2*	ENSOARG00020003758
13	56473081	56516091	*KCNQ2*	ENSOARG00020003802
13	56544051	56556529	*CHRNA4*	ENSOARG00020004285
13	56588370	56598084	*ARFGAP1*	ENSOARG00020004787
13	56619433	56622851	*BIRC7*	ENSOARG00020005025
13	56632628	56644231	*YTHDF1*	ENSOARG00020005076
13	56805894	56821189	*SLC17A9*	ENSOARG00020005151
13	56826542	56832218	*GID8*	ENSOARG00020005239
13	56887526	56904123	*TCFL5*	ENSOARG00020005326

Chr	Gene Start (bp)	Gene End (bp)	Gene Name	Ensembl Gene ID
13	56905051	56923339	*COL9A3*	ENSOARG00020005388
13	56926591	56933668	*OGFR*	ENSOARG00020005520
13	56936611	56940173	*MRGBP*	ENSOARG00020005556
17	40651758	40707383	*NUDT6*	ENSOARG00020010377
17	40707674	40762443	*FGF2*	ENSOARG00020010451

Table S3. GO terms and KEGG pathways enriched ($p < 0.05$) based on ROH islands.

ID	Term	P-Value	Input number
oas05016	Huntington disease	0.000899	6
oas04390	Hippo signaling pathway	0.001507	5
oas05226	Gastric cancer	0.001721	5
oas03013	RNA transport	0.002496	5
oas05218	Melanoma	0.006674	3
oas05224	Breast cancer	0.008981	4
oas04080	Neuroactive ligand-receptor interaction	0.013057	6
oas04151	PI3K-Akt signaling pathway	0.017555	6
oas05033	Nicotine addiction	0.020279	2
oas04919	Thyroid hormone signaling pathway	0.025622	3
oas05030	Cocaine addiction	0.027889	2
oas04110	Cell cycle	0.030068	3
oas04068	FoxO signaling pathway	0.035523	3
oas05213	Endometrial cancer	0.038738	2
oas04550	Signaling pathways regulating pluripotency of stem cells	0.038759	3
oas04120	Ubiquitin mediated proteolysis	0.042834	3
oas00190	Oxidative phosphorylation	0.044238	3
oas05217	Basal cell carcinoma	0.047131	2
oas04137	Mitophagy - animal	0.04838	2
GO:0003677	DNA binding	0.030167	1
GO:0005507	copper ion binding	0.030167	1
GO:0045766	positive regulation of angiogenesis	0.030167	1
GO:0000981	DNA-binding transcription factor activity, RNA polymerase II-specific	0.030167	1
GO:0070374	positive regulation of ERK1 and ERK2 cascade	0.04002	1
GO:0005739	mitochondrion	0.04002	1
GO:0030308	negative regulation of cell growth	0.04002	1

Table S4. Candidate gene enrichment based on human GWAS and TWAS results.

Gene	ENSG ID	Associated Diseases	Main Associated Tissues
FGF9	ENSG00000102678.6	Vascular/heart problems, High blood pressure	Adipose Subcutaneous,Adrenal Gland,Artery Coronary,Lung,Testis
BMPR1B	ENSG00000138696.10	heart/cardiac problem	Testis
EFNB3	ENSG00000108947.4	hypertension	Brain Caudate basal ganglia,Brain Putamen basal ganglia,Colon Transverse,Ovary,Brain Substantia nigra
LGSN	ENSG00000146166.16	Blood clot, DVT, bronchitis, emphysema, asthma, rhinitis, eczema, allergy	Whole Blood, Lung, Small Intestine Terminal Ileum, Spleen,Testis
OCA2	ENSG00000104044.15	hepatitis	Muscle Skeletal
SPATA5	ENSG00000145375.7	Blood clot, DVT, bronchitis, emphysema, asthma, rhinitis, eczema, allergic rhinitis or eczema	Adipose Subcutaneous, Esophagus Muscularis, Ovary
HERC2	ENSG00000128731.15	Senile cataract	Brain Putamen basal ganglia,Breast Mammary Tissue, Ileum,Spleen,Stomach,Testis,
MICU2	ENSG00000165487.13	hypertension	Artery Aorta,Brain Nucleus accumbens basal ganglia, Esophagus Muscularis,Heart Atrial Appendage
OCA2	ENSG00000104044.15	hepatitis	Muscle Skeletal

Gene	ENSG ID	Associated Diseases	Main Associated Tissues
SPATA5	ENSG00000145375.7	Blood clot, DVT, bronchitis, emphysema, asthma, rhinitis, eczema, allergy, allergic rhinitis or eczema	Adipose Subcutaneous,Adipose Visceral Omentum,Artery Aorta,Brain Nucleus accumbens basal ganglia,Esophagus Muscularis,Heart Left Ventricle,Ovary,Pituitary,Uterus,Minor Salivary Gland,Joint Tissues
GFRA3	ENSG00000146013.10	Atrial fibrillation	Adipose Visceral Omentum,Testis,Vagina
CDC23	ENSG00000094880.10	Male-specific factors - Hair/balding	Brain Cerebellar Hemisphere,Thyroid
CDC25C	ENSG00000158402.18	Atrial fibrillation	Whole Blood,Esophagus Muscularis,Liver,Lung,Skin Sun Exposed Lower leg,Small Intestine Terminal Ileum
